# Activation of endoplasmic reticulum stress response by enhanced polyamine catabolism is important in the mediation of cisplatin-induced acute kidney injury

**DOI:** 10.1371/journal.pone.0184570

**Published:** 2017-09-08

**Authors:** Kamyar Zahedi, Sharon Barone, Christina Destefano-Shields, Marybeth Brooks, Tracy Murray-Stewart, Matthew Dunworth, Weimin Li, Joanne R. Doherty, Mark A. Hall, Roger D. Smith, John L. Cleveland, Robert A. Casero, Manoocher Soleimani

**Affiliations:** 1 Departments of Medicine, University of Cincinnati College of Medicine, Cincinnati, OH, United States of America; 2 Center on Genetics of Transport, University of Cincinnati College of Medicine, Cincinnati, OH, United States of America; 3 Research Services, Veterans Affairs Medical Center, Cincinnati, OH, United States of America; 4 The Sidney Kimmel Comprehensive Cancer Center, Johns Hopkins University School of Medicine, Baltimore, Maryland, United States of America; 5 Department of Tumor Biology, Moffitt Cancer Center and Research Institute, Tampa, FL, United States of America; 6 Department of Cancer Biology, The Scripps Research Institute, Jupiter, FL, United States of America; 7 Department of Pathology and Laboratory Medicine, University of Cincinnati College of Medicine, Cincinnati, OH, United States of America; ENEA Centro Ricerche Casaccia, ITALY

## Abstract

Cisplatin-induced nephrotoxicity limits its use in many cancer patients. The expression of enzymes involved in polyamine catabolism, spermidine/spermine N^1^-acetyltransferase (SSAT) and spermine oxidase (SMOX) increase in the kidneys of mice treated with cisplatin. We hypothesized that enhanced polyamine catabolism contributes to tissue damage in cisplatin acute kidney injury (AKI). Using gene knockout and chemical inhibitors, the role of polyamine catabolism in cisplatin AKI was examined. Deficiency of SSAT, SMOX or neutralization of the toxic products of polyamine degradation, H_2_O_2_ and aminopropanal, significantly diminished the severity of cisplatin AKI. *In vitro* studies demonstrated that the induction of SSAT and elevated polyamine catabolism in cells increases the phosphorylation of eukaryotic translation initiation factor 2α (eIF2α) and enhances the expression of binding immunoglobulin protein BiP/GRP78) and CCAAT-enhancer-binding protein homologous protein (CHOP/GADD153). The increased expression of these endoplasmic reticulum stress response (ERSR) markers was accompanied by the activation of caspase-3. These results suggest that enhanced polyamine degradation in cisplatin AKI may lead to tubular damage through the induction of ERSR and the consequent onset of apoptosis. In support of the above, we show that the ablation of the SSAT or SMOX gene, as well as the neutralization of polyamine catabolism products modulate the onset of ERSR (e.g. lower BiP and CHOP) and apoptosis (e.g. reduced activated caspase-3). These studies indicate that enhanced polyamine catabolism and its toxic products are important mediators of ERSR and critical to the pathogenesis of cisplatin AKI.

## Introduction

Cisplatin, a platinum based compound, is a commonly used and highly effective chemotherapeutic agent utilized for the treatment of a variety of solid tumors [1, 2]. The principal mode of cisplatin anti-tumor activity is via the formation of DNA–protein and DNA–DNA adducts [3, 4]. The non-repairable cisplatin-induced DNA damage results in the inhibition of tumor cell division and induction of apoptosis. Despite its effectiveness, cisplatin usage is limited due its ototoxic and nephrotoxic side effects. More than 25% of patients treated with cisplatin develop renal failure and have to discontinue treatment [1, 5]. The molecular mechanisms of cisplatin nephrotoxicity are not completely elucidated and it is most likely a process that depends on the activation of multiple pathways and mechanisms.

**Fig 1:**
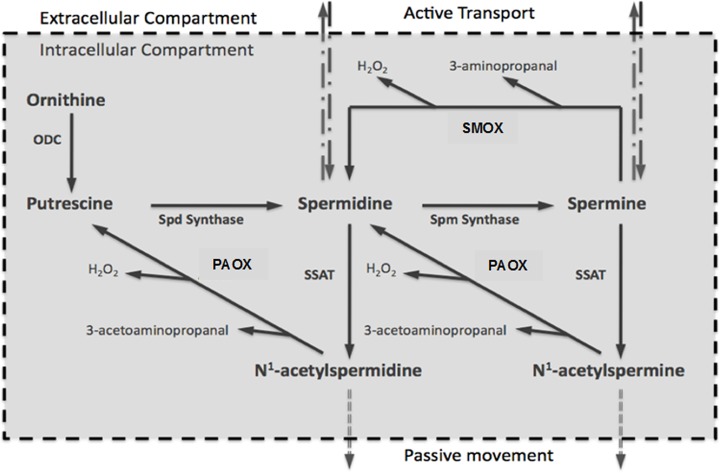
Depiction of polyamine synthetic and catabolic reactions. This schematic indicates that the oxidation of acetylated polyamines and via APAOX or SMOX, respectively, leads to the generation of cytotoxic molecules (H_2_O_2_ and aminoaldehydes).

Polyamines are aliphatic cations that play important roles in the regulation of DNA structure, DNA/protein and protein/protein interactions, as well as the scavenging of free radicals [6–9]. They are indispensable in the maintenance of genomic integrity and in the regulation of cell growth and viability [6–10]. Cellular levels of polyamines are tightly regulated through their synthesis and degradation **(Fig 1)**. Polyamine synthesis is initiated by ornithine decarboxylase (ODC) mediated decarboxylation of ornithine to form putrescine (Put). Sequential enzymatic addition of aminopropyl groups to Put and spermidine (Spd); respectively, leads to the formation of Spd and spermine (Spm). Polyamines are degraded through their back-conversion via the spermidine/spermine N^1^-acetyltransferase/N^1^-acetylpolyamine oxidase (SSAT/PAOX) cascade, and direct oxidation of Spm by spermine oxidase (SMOX). Oxidation of acetyl-Spm and acetyl-Spd by PAOX and Spm by SMOX generates toxic molecules such as H_2_O_2_ and aminoaldehydes [11]. Polyamines are present in significant intracellular concentrations (mM range); therefore, substantial concentrations of H_2_O_2_ and aminoaldehydes (e.g. 3-aminopropanal, 3-acetoaminopropanal and acreloin) can be produced via their catabolism [12]. While H_2_O_2_ through generation of hydroxyl radicals causes DNA lesions [13, 14], both aminoaldehydes and H_2_O_2_ disrupt the integrity of lysosomal and mitochondrial membranes, causing further cell injury [15–18]. The expression of SSAT in cultured cells leads to increased SMOX expression, alterations in polyamine homeostasis, DNA damage, mitochondrial dysfunction, growth arrest and apoptosis [19].

Catabolism of polyamines (Spd and Spm) is enhanced in the kidney, brain, liver, stomach, colon and heart in response to ischemic reperfusion (I/R), toxic, septic or traumatic insults [20–25]. In addition, expression of polyamine catabolic enzymes increases in, and is associated with the remote organ dysfunction following an initial injury (e.g. liver damage following AKI) [26]. The ablation of the SSAT gene or inhibition of polyamine oxidases by MDL72527 reduces the severity of tissue damage caused by I/R, toxic or septic injuries [23, 27–29].

The increased polyamine catabolism can cause tissue/organ damage consequent to decreased levels of radical-scavenging natural polyamines and/or generation of reactive oxygen molecules (e.g. H_2_O_2_) and aldehydes (e.g. 3-aminopropanal and acrolein) [20, 30–32]. The role of by-products of polyamine catabolism in the mediation of tissue injury in cerebral ischemia has been previously examined. The aforementioned studies indicate that 3-aminopropanal and acrolein contribute to an increased aldehyde load and cause tissue damage via disruption of mitochondrial function and lysosome membrane damage [30, 33, 34]. These molecules are also important inducers of Endoplasmic reticulum stress response (ERSR) and apoptosis [35, 36].

ERSR is the biological rescue response to the accumulation of misfolded proteins in cells [37, 38]. The role of ERSR in the pathophysiology of I/R, radiocontrast medium, tunicamycin and cisplatin induced AKI is well documented [37, 38]. Reactive oxygen species (ROS) and aldehydes that are among the main molecular mediators of tissue damage in all of the above injuries are also known to be important inducers of ERSR-mediated apoptosis [39, 40].

Using genetically engineered mice lacking SSAT or SMOX (SSAT-KO or SMOX-KO), and through neutralization of toxic products of polyamine degradation we tested the following: 1) whether or not SSAT and SMOX expression levels increase in response to cisplatin treatment; 2) is cisplatin-induced AKI in part mediated via enhanced activity of polyamine catabolic enzymes and through generation of toxic products of polyamine degradation (e.g. H_2_O_2_ and aminoaldehydes); and 3) does activation of polyamine catabolism induces ERSR, a pathway that is critical to the mediation of cell injury, tissue damage and organ dysfunction.

## Materials and methods

### Reagents

All chemicals and reagents were purchased from Sigma-Aldrich (St. Louis, MO) unless otherwise indicated. Oligonucleotides were purchased from ThermoFisher Scientific (Carlsbad, CA). The following antibodies were used in this study: Rabbit anti-β-actin (Santa Cruz Biotech, Santa Cruz, CA), Rabbit anti-pro and cleaved Caspase 3 (H-277, Santa Cruz Biotech, Santa Cruz, CA), Rabbit anti-cleaved caspase 3 (Sigma-Aldrich, St Louis, MO), Rabbit anti-CHOP/GADD153 (Santa Cruz Biotech, Santa Cruz, CA) and Goat anti BiP/GRP78 (Santa Cruz Biotech, Santa Cruz, CA). Anti-hypusinated-eukaryotic translation initiation factor 5A (eIF5A) was kind gift of Dr. R.G. Mirmira, Indiana School of Medicine (Indianapolis, Indiana). Anti SMOX antibody was generated by Dr. R. A. Casero Jr. All secondary antibodies were purchased from ThermoFisher Scientific.

Generation and genotyping of SSAT- and SMOX-KO mice. SSAT deficient (SSAT-KO) mice were the kind gift of Dr. Carl W. Porter. Their generation and genotyping protocol has been previously described [41, 42].

SMOX-deficient mice were generated through elimination of exons IV, V, and VI by homologous recombination. The latter disrupts the FAD binding region (exon V) of SMOX. The targeting vector for the *Smox* knockout mouse was made using standard recombinant methods. Briefly, the murine *Smox* gene was subcloned into the pBluescript SK(-) vector. A neomycin cassette was introduced into the *Smox* locus; the resulting insertion led to the excision of exons IV, V, and VI **(S1A Fig)**. The vector was linearized and transfected into 129S6/SvEvTac ES cells. ES cells that had undergone homologous recombination were then selected and subjected to Southern blot analysis (**S1B Fig**). Chimeras were bred onto C57Bl/6J wild-type mice to generate F1 offspring. Offspring were genotyped as described **(S1 Table)**. Mice were back crossed to wild-type C57Bl/6J (Jackson labs) for 10 generations. Heterozygotes were then intercrossed to produce *Smox*-KO mice.

### Mouse model of cisplatin induced AKI

Studies outlined in this section were designed using ARRIVE guide lines and approved by University of Cincinnati’s Institutional Animal Care and Use Committee (IACUC, protocol number 04020901). Wild type (Wt) and genetically modified mice (n = 8/treatment group) were administered a single intraperitoneal (i.p.) injection of vehicle (saline) or cisplatinum (20mg/Kg). Studies examining the effect of neutralization of polyamine catabolites on the severity of cisplatin AKI were performed in Wt mice (n = 6/treatment group) as outlined above. Animals were given daily i.p. injection of phenelzine (30mg/kg/day) or a combination of PEG-catalase (50units/g/day) and N-2-MPG (100mg/kg/day) for the duration of the studies (96 hrs). Animals were euthanized by an over dose (150μl) of Euthazol (390mg Sodium pentobarbital and 50mg phenytoin/100 ml) and processed to obtain the needed experimental specimens including serum for the measurement of serum creatinine, kidneys for extraction of RNA and protein as well as measurement of polyamines and polyamine pathway enzyme levels. Kidney samples were also harvested fixed in paraformaldehyde and preserved in 70% ethanol for histology and immunofluorescence microscopy studies.

### Tissue culture studies

Using HEK-SSAT-TREX cells that express high levels of SSAT upon exposure to tetracycline[32], we determined the effect of enhanced production of this enzyme on tissue polyamine catabolism, induction of ERSR and onset of apoptosis. Briefly, HEK_SSAT_TREX cells (seeded at 2x10^6^/100mm plate) were allowed to stabilize for 48 hours, treated with tetracycline (10μg/ml) and harvested at timed intervals (3, 6, 15, 24, 48 and 72 hours). Time matched vehicle treated cells served as controls. Cells were harvested and snap frozen in liquid nitrogen. Frozen cells were processed for measurement of polyamines and the activity of polyamine pathway enzymes, or processed for protein extraction.

### Assessment of renal function

Serum creatinine levels were measured using a commercially available kit (Bioassay Systems, Hayward, CA) following the manufacturer’s instructions.

### Histopathology of kidney

The severity of renal damage was determined by examining the cortical and corticomedullary regions of the kidney for tubular dilatation, cast formation, and edema.

### Measurement of kidney ODC, SSAT and polyamine levels

Renal activity of SSAT and ODC, as well as polyamine pools were analyzed as described previously [22, 28].

### RNA extraction and Northern blot analysis

RNA was extracted using Tri-Reagent (MRC, Cincinnati, OH) and subjected to Northern blot analysis as described previously [28].

### Preparation of cell and kidney protein extracts and Western blot analysis

Cell extracts were prepared by lysing the frozen cell pellets in RIPA buffer (Thermo Scientific, Rockford, IL), supplemented with protease and phosphatase inhibitors (Thermo Scientific). Kidney extracts were prepared using T-PER buffer (Thermo Scientific), supplemented with protease and phosphatase inhibitors (Thermo Scientific). Protein concentrations were determined using BCA assay kit (Thermo Scientific) subjected to Western blot analysis as described previously [28].

### Statistical analysis

The significance of differences between mean values±SEM of multiple samples will be examined using ANOVA. A *“P”* value of <0.05 was considered statistically significant.

## Results

### Polyamine catabolism is enhanced in the kidneys of cisplatin-treated mice

**Fig 2:**
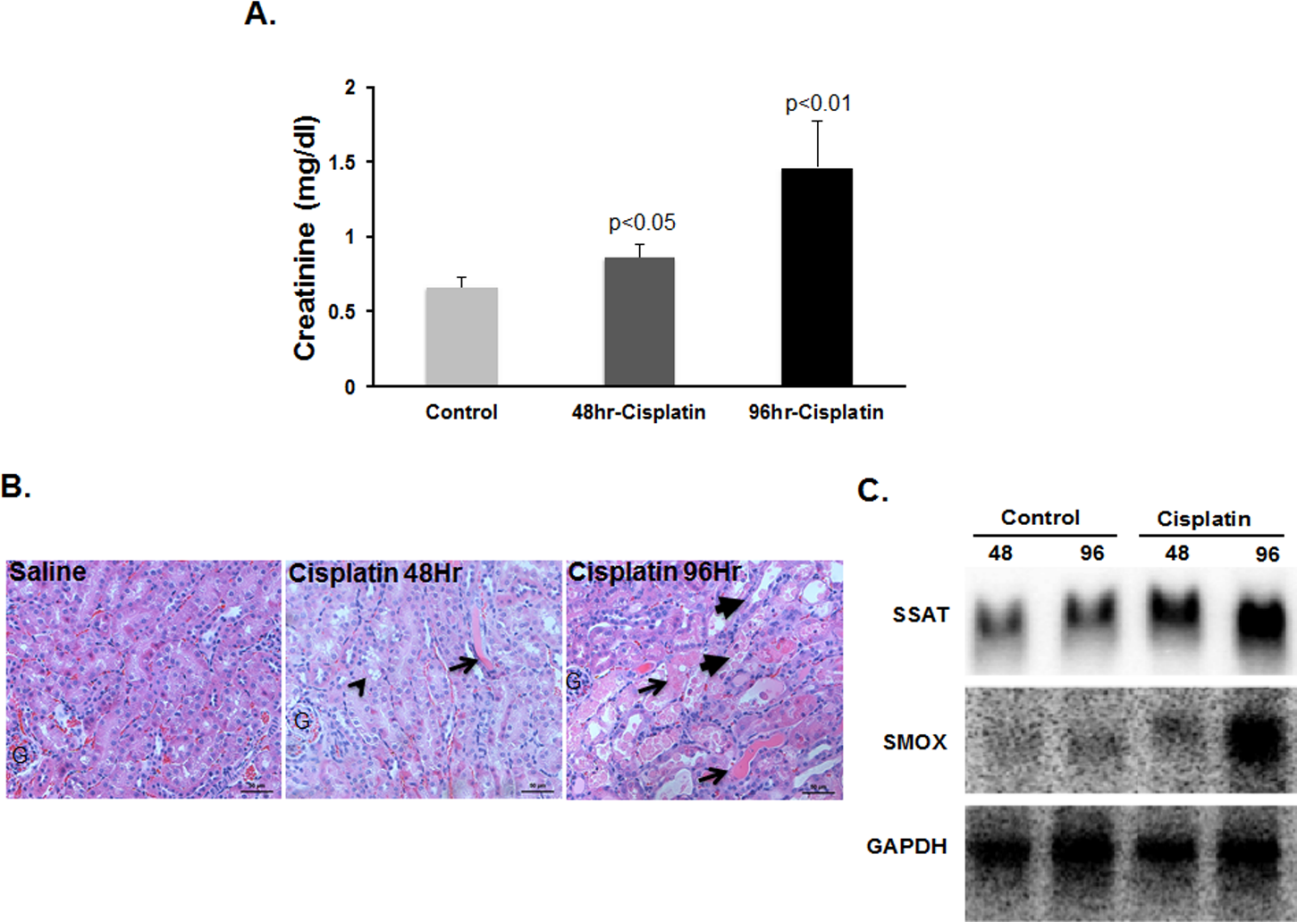
Effect of cisplatin treatment on renal function and structure and the expression of polyamine catabolic enzymes. A & B) Administration of cisplatin (20mg/kg) led to significantly increased serum creatinine levels tubular injury ranging from mild (48 hours) to severe (96 hours) post-treatment (vacuolization, small arrow head; cast, small arrows; sloughed cells and damaged tubules, large arrows). C) Expression of SSAT and SMOX mRNA levels increase in kidneys of mice treated with cisplatin.

**Fig 3:**
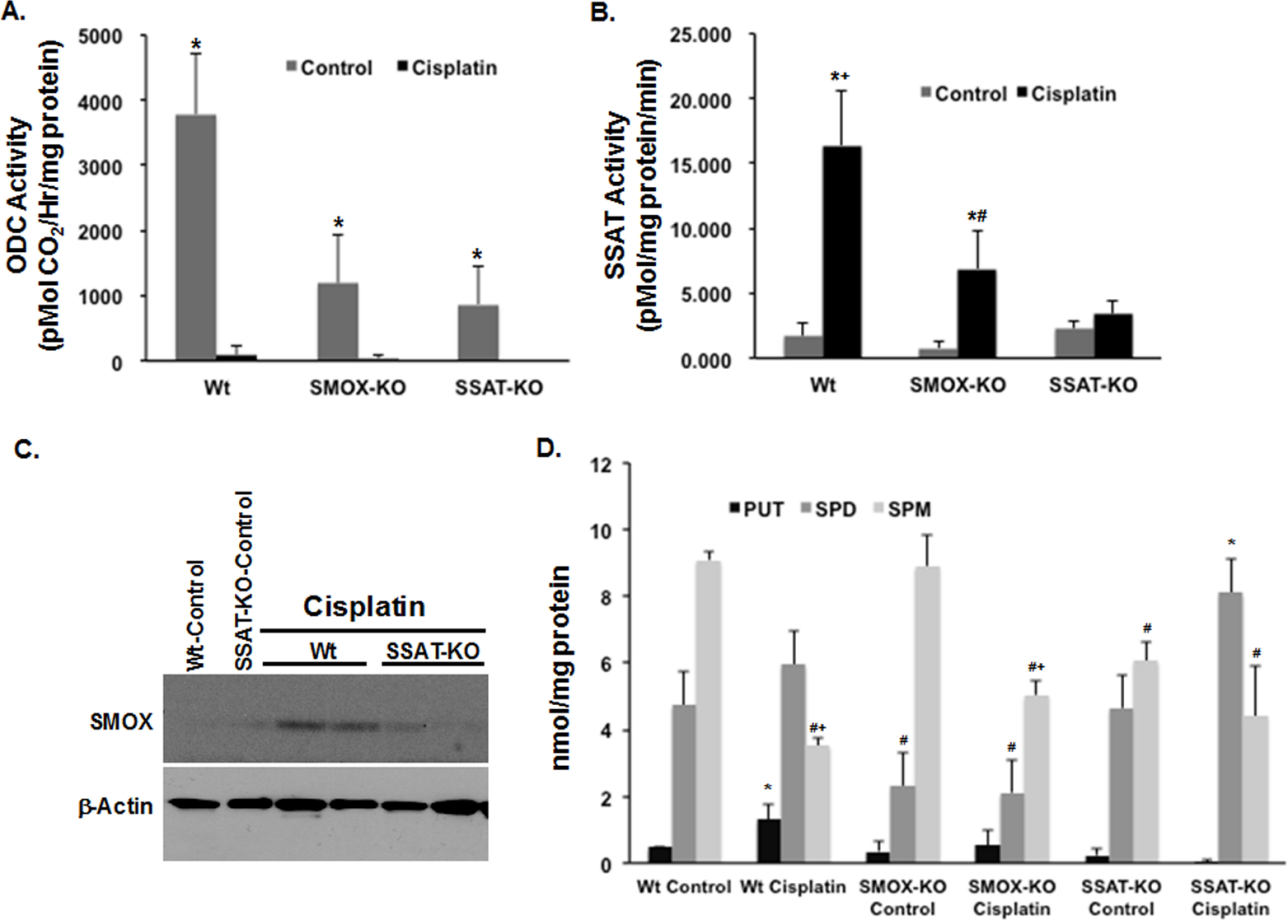
Effect of SSAT and SMOX ablation on cisplatin AKI induced changes in ODC, SSAT and polyamine levels. A) The activity of ODC is approximately 2-fold lower in saline treated SSAT-KO and SMOX-KO mice compared to their Wt littermates (p<0.05); however, the reduction in ODC activity in all 3 groups subsequent to cisplatin treatment is of a similar magnitude and significant. (*Denotes significantly higher enzymatic activity in control vs. cisplatin-treated animals). B) Renal SSAT activity is similar in saline treated Wt and SMOX-KO mice. The SSAT activity is significantly elevated in Wt mice treated with cisplatin compared to those treated with saline. (*Denotes significantly higher enzymatic activity in cisplatin-treated vs. control animals. ^+^Denotes a significant increase in cisplatin-treated Wt compared to similarly treated SMOX-KO and SSAT-KO mice. ^#^Denotes significant increase in cisplatin-treated SMOX-KO to SSAT-KO mice). C) SMOX protein levels are elevated in the kidneys of cisplatin-treated Wt animals compared to saline treated and cisplatin treated SSAT-KO mice. D) Examination of tissue polyamine levels reveals that the kidney Put content increases significantly in Wt but not SMOX- and SSAT-KO mice after cisplatin treatment. The Spd content of the kidneys only marginally increases in Wt and SSAT-KO mice after cisplatin treatment. Examination of Spm levels indicates that their reduction is significantly greater in the Wt mice than SMOX-KO and SSAT-KO animals. (*Denotes significantly increased content in cisplatin-treated vs saline-treated animals of the same genotype. ^+^Denotes a significant decrease in tissue content in cisplatin-treated animals compared to saline-treated animals of the same genotype. ^#^Denotes a significant decrease in tissue content compared to saline-treated Wt mice).

In order to determine if polyamine catabolism is enhanced in response to treatment with cisplatin, male C57BL/6 mice were given a single i.p. injection of cisplatin (20mg/kg). Increased serum creatinine levels **(Fig 2A)** and tubular damage **(Fig 2B)** confirmed the induction of cisplatin AKI. Mice treated with saline were used as controls. Cisplatin-treated and control animals were sacrificed 48 and 96 hours after treatment. Northern blot analysis of kidney RNA from control and cisplatin-treated mice revealed significant increase in the expression of the polyamine pathway catabolic enzymes, SSAT and SMOX transcripts, at 48 and 96 hours post-cisplatin treatment **(Fig 2C)**. The increase in polyamine catabolism in cisplatin AKI was further confirmed by the determination of renal polyamine levels and activity of polyamine pathway enzymes. These results demonstrate the presence of elevated SSAT activity (*P*<0.01), reduced ODC activity (*P*<0.05), and increased expression of SMOX protein in response to treatment with cisplatin **(Fig 3A–3C)**. Assessment of kidney polyamine levels revealed that cisplatin treatment leads to increased accumulation of Put (*P*<0.01); in addition to a greater than 62% reduction (*P*<0.01) in kidney Spm at 96 hours post cisplatin administration **(Fig 3D)**.

### Ablation of SSAT and SMOX protects against cisplatin AKI

**Fig 4:**
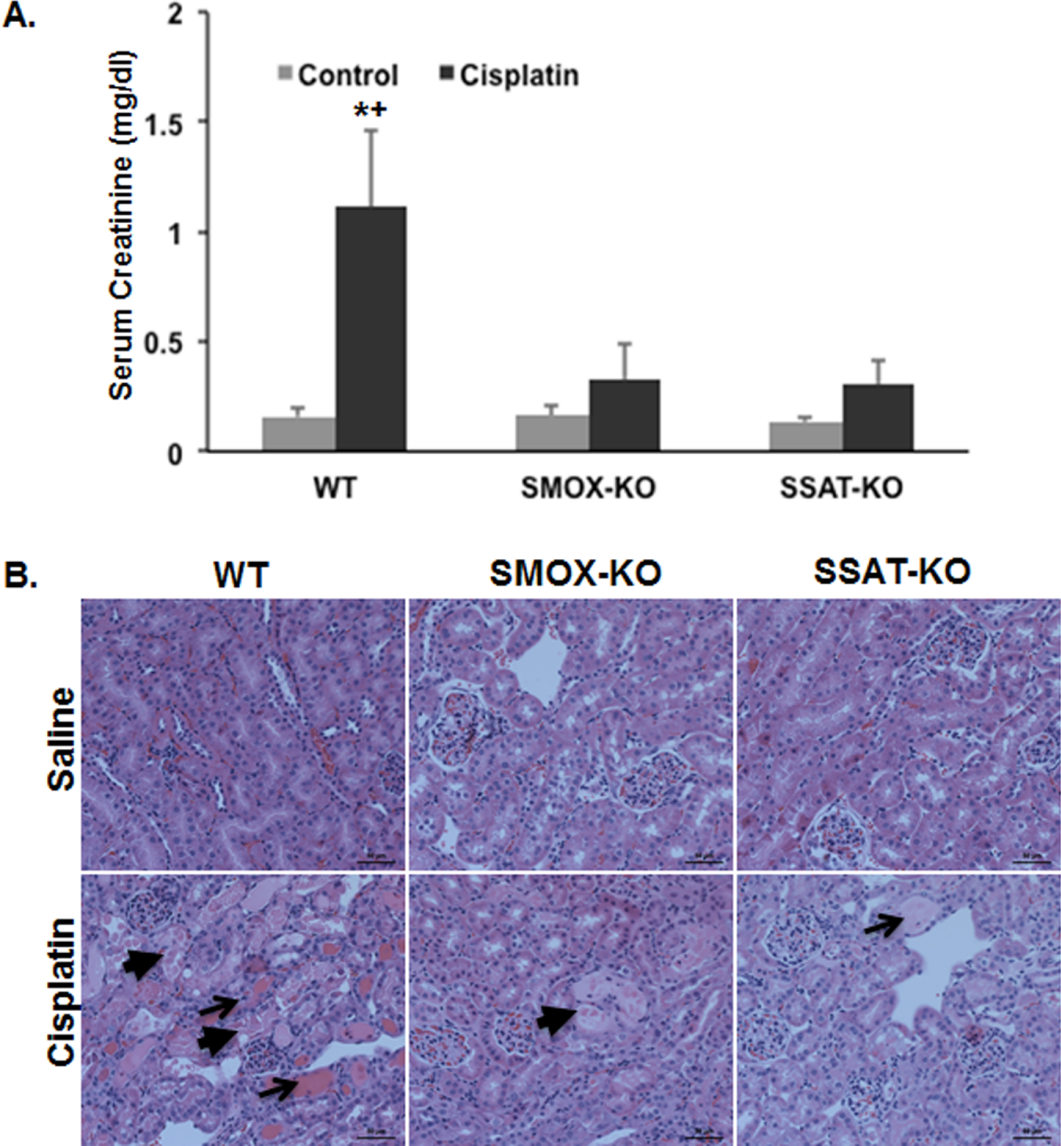
Effect of SSAT and SMOX ablation on cisplatin AKI. Ablation of SSAT and SMOX genes reduces the loss of renal function (A) and protects against renal tubular injury caused by cisplatin treatment (B). (*Denotes significant increase in creatinine levels in cisplatin-treated vs. control animals. ^**+**^Indicates significant increase in cisplatin-treated Wt compared to similarly treated SMOX-KO and SSAT-KO animals.

In order to establish the role of polyamine catabolism in the mediation of cisplatin AKI, the extent of the loss of renal function and severity of tubular damage was compared in the Wt, SSAT-KO and SMOX-KO mice. Our results indicate that serum creatinine levels of saline-treated Wt, SSAT-KO and SMOX-KO mice were similar; however, the serum creatinine levels of Wt mice were significantly higher than both SSAT-KO (*P*<0.01) and SMOX-KO (*P*<0.01) mice at 96 hours post-cisplatin treatment **(Fig 4A)**. Examination of the renal histology also revealed that compared to the kidneys of Wt mice, kidneys of SSAT-KO and SMOX-KO were significantly protected against tubular damage by cisplatin treatment **(Fig 4B)**.

Examination of the effect of cisplatin treatment on the activity of polyamine pathway enzymes, ODC and SSAT, revealed that increases in SSAT activity were only significant in Wt animals (*P*<0.05). While the activity of ODC was nearly 2-fold lower in saline-treated SSAT- and SMOX-KO mice compared to their Wt littermates, the reduction in ODC activity in all 3 genotypes subsequent to cisplatin treatment was of a similar magnitude **(**approximately 50%; **Fig 3A)**. Measurement of SSAT activity also revealed that the increase in the activity of SSAT after cisplatin treatment in SMOX-KO was less than half of that of Wt mice **(***P* = 0.066, **Fig 3B)**. In addition, comparison of kidney levels of SMOX protein revealed that the expression of this protein is significantly higher in cisplatin-treated Wt mice than saline-treated control mice or cisplatin-treated SSAT-KO mice **(Fig 3C).** Examination of tissue polyamine levels revealed that the after cisplatin treatment kidney Put content increased significantly in Wt but not in SMOX- or SSAT-KO mice **(Fig 3D)**. The Spd content of the kidneys increased in Wt (*P*<0.01) and SSAT-KO (*P*<0.01) but not SMOX mice after cisplatin treatment **(Fig 3C)**. Examination of Spm levels indicates that their reduction in cisplatin treated vs control animals is of a greater magnitude in the Wt mice (62%; *P*<0.01) than SMOX-KO (43%; *P*<0.01) and SSAT-KO (28%; *P =* 0.102) animals.

### Neutralization of toxic products of polyamine degradation significantly reduces the severity of cisplatin AKI in Wt mice

**Fig 5:**
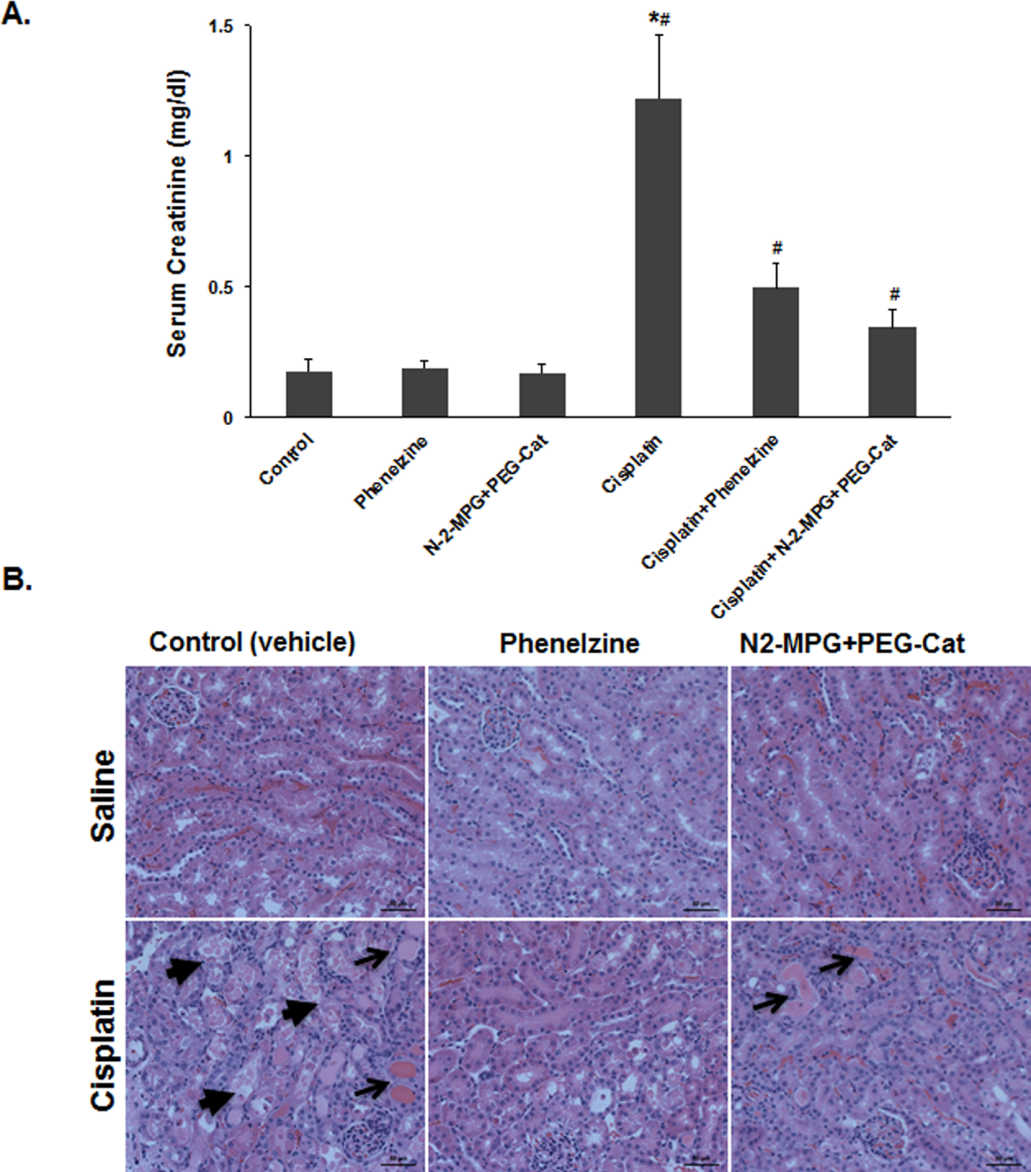
Effect of neutralization of toxic products of polyamine degradation on cisplatin AKI in Wt mice. A) The serum creatinine levels of saline-treated animals receiving vehicle, PEG-Cat/N-2-MPG or PLZ were similar. While serum creatinine levels were significantly lower in cisplatin-injected animals treated with a combination of PEG-Cat (50units/g/day) and N-2-MPG (100mg/kg/day) or PLZ (30mg/kg/day). B) The renal histology of saline-treated animals receiving vehicle, PEG-Cat/N-2-MPG or PLZ were normal. However, the tubules were significantly protected in cisplatin-injected animals treated with a combination of PEG-Cat/N-2-MPG or PLZ. (*Denotes a significant increase in serum creatinine of cisplatin-treated mice compared to similarly treated animals given PEG-Cat/N-2-MPG or PLZ. ^#^ Denote significant increases in the serum creatinine levels of cisplatin-treated compared to saline-treated mice in corresponding treatment groups).

Aminoaldehydes and H_2_O_2_ can cause cell injury through induction of DNA damage and disruption of lysosomes and mitochondria[15–18]. Since polyamines are present at mM concentrations in the cell, comparable levels of H_2_O_2_ and aminoaldehydes can be generated through their catabolism [12]. The latter can make the catabolism of polyamines an important source of these toxic molecules. In order to ascertain the role of these molecules in the mediation of cisplatin AKI, the effect of treatment with reagents that degrade H_2_O_2_ (e.g. cell permeable polyethyleneglycol conjugated catalase; PEG-Cat) and compounds that neutralize (e.g. N-2-mercaptopropionyl glycine, N-2-MPG) or sequester (e.g. phenelzine; PLZ) aminoaldehydes and acrolein on the severity of renal dysfunction and tubular damage caused by cisplatin was determined. The serum creatinine levels and renal histology of saline-treated animals receiving vehicle, PEG-Cat/N-2-MPG or PLZ were similar **(Fig 5A)**. However, serum creatinine levels were significantly lower **(***P*<0.01; **Fig 5A)** and the tubules were significantly protected **(Fig 5B)** in cisplatin-injected animals treated with a combination of PEG-Cat (50units/g/day) and N-2-MPG (100mg/kg/day) or PLZ (30mg/kg/day).

### Expression of SSAT in cultured cells activates the ERSR and enhances apoptosis

**Fig 6:**
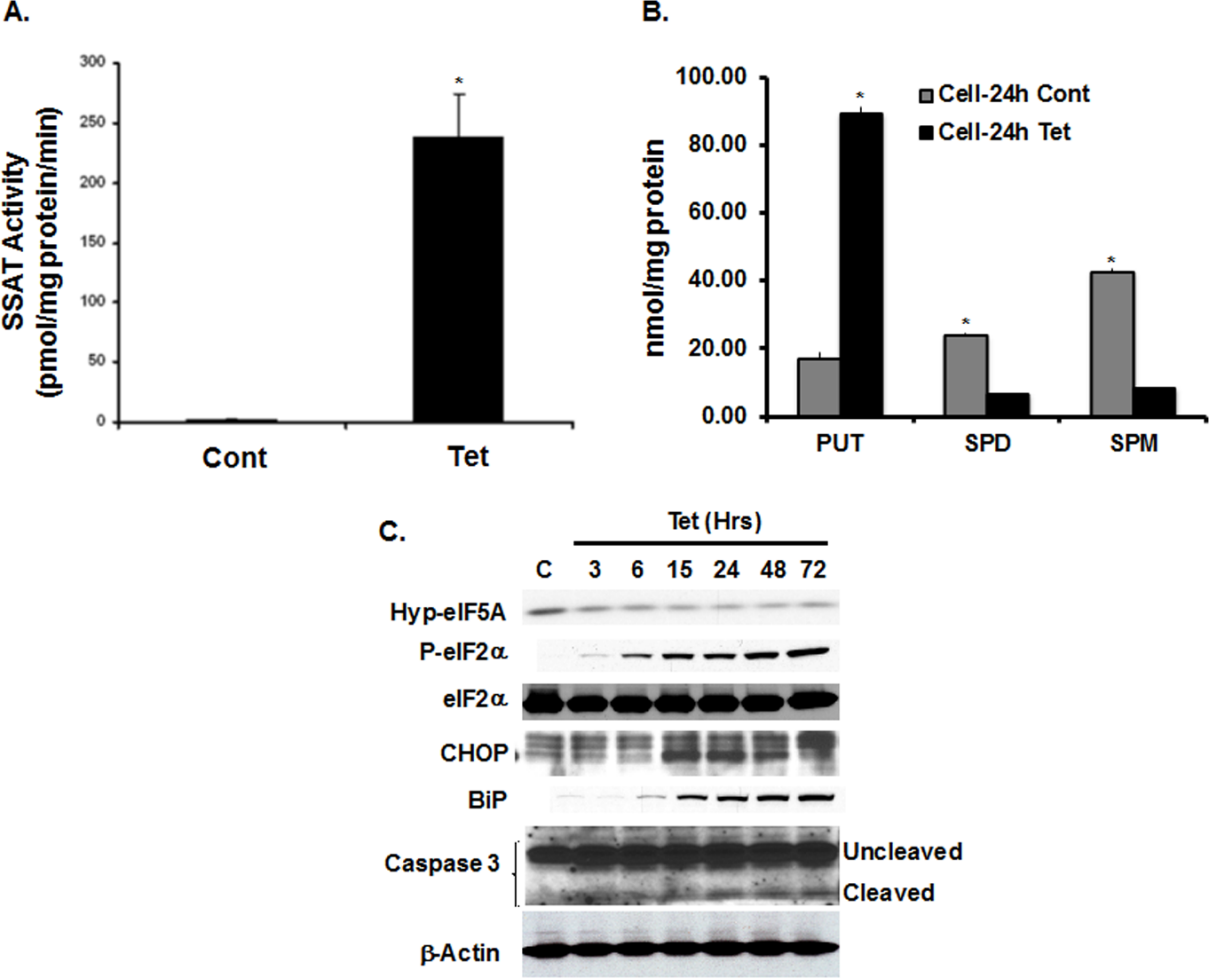
Overexpression of SSAT in cultured cells: impact on ERSR and apoptosis. A & B) Treatment of HEK-SSAT-Trex cells with tetracycline led to the induction of SSAT a significant elevation in Put and reductions in Spd and Spm levels. C) Western blot analyses indicate that the induction of SSAT leads to a reduction in hypusinated-eIF5A levels, a time-dependent transient increase in p-eIF2α, BiP and CHOP levels. Increased activated caspase3 levels were also detected in samples from 48 to 72 hours post Tetracycline-induction of SSAT expression. The results are representative of 3 independent experiments (* Denotes *P*<0.01).

Using HEK cells (HEK-SSAT-Trex) that express SSAT upon exposure to tetracycline[32, 43], we examined the effect of enhanced expression of SSAT on the induction of ERSR *in vitro*. Treatment of HEK-SSAT-Trex cells with tetracycline led to the induction of SSAT **(Fig 6A)**, a significant elevation in Put and reductions in Spd and Spm levels **(Fig 6B)**. Western blot analyses indicate that the levels of hypusinated-eIF5A (hypusination of eIF5A is necessary for its function during protein synthesis) are reduced in SSAT over-expressing HEK cells **(Fig 6C)**. Our results indicate that the induction of SSAT also leads to a time-dependent transient increase in cellular levels of p-eIF2α, BiP/GRP78 and the pro-apoptotic protein, CHOP **(Fig 6C)**. Increased activated caspase 3 levels were also detected in cell extracts from 48 to 72 hours after Tetracycline-induction of SSAT **(Fig 6C)**. Collectively, these results indicate that enhanced catabolism of polyamines as a result of enhanced SSAT expression can lead to the induction of ERSR and onset of apoptosis.

### Polyamine catabolism in cisplatin AKI mediates the induction of ERSR and the onset of apoptosis

**Fig 7:**
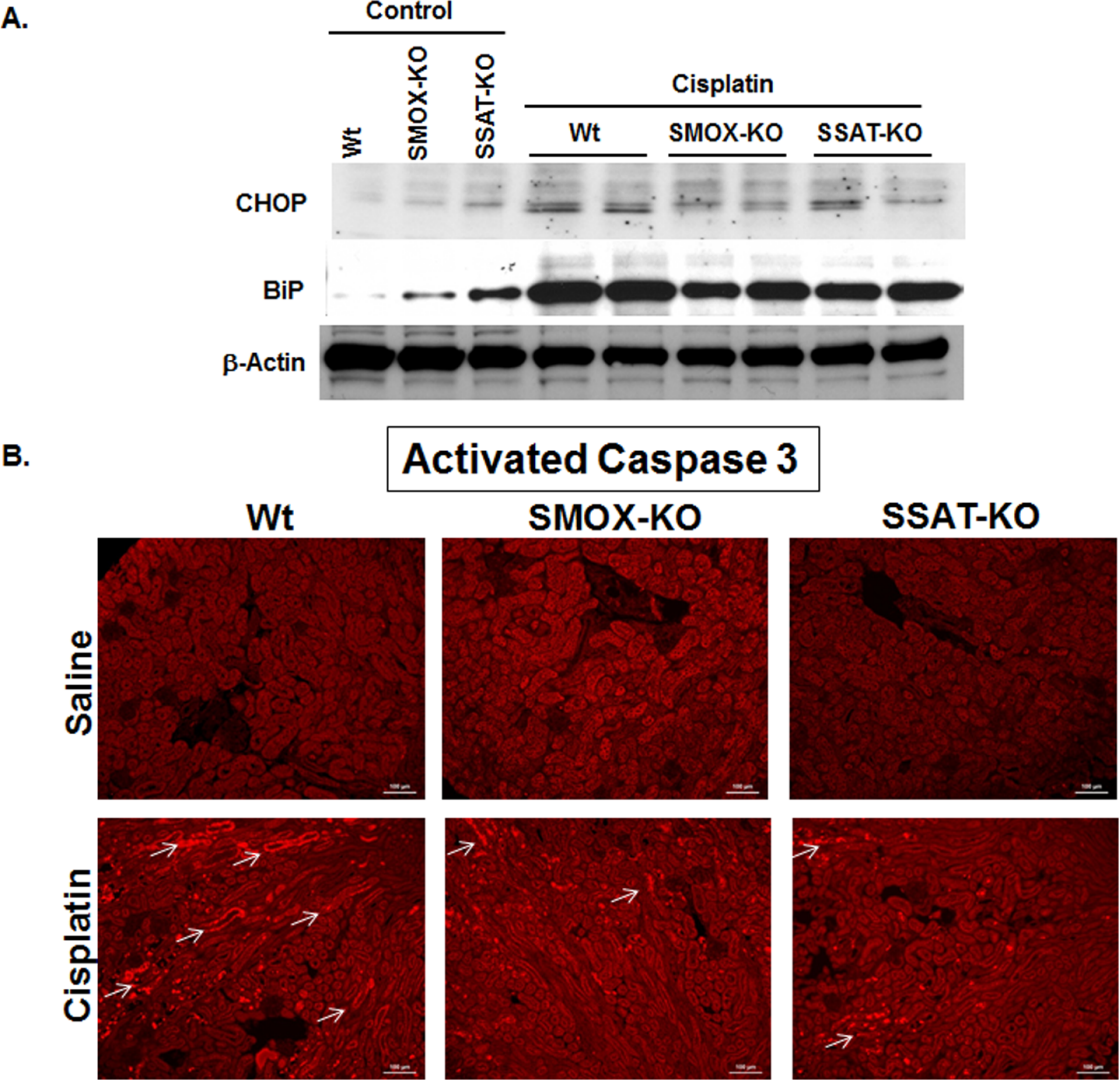
The consequence of SSAT and SMOX ablation on the induction of ERSR and onset of apoptosis. The effect of modulation of polyamine catabolism on the activation of ERSR and onset of apoptosis in cisplatin AKI was examined. A) The levels of BiP and CHOP were similar in the control samples from all 3 genotypes. The expression of BiP and CHOP increased in all three genotypes after cisplatin treatment; however, the BiP and CHOP expression levels were reduced in cisplatin treated SMOX-KO and SSAT-KO mice compared to their cisplatin-treated Wt counterparts. The results are representative of 3 independent experiments. B) The levels of activated caspase 3 (arrows) were also significantly elevated in the kidneys of Wt compared to SSAT-KO and SMOX-KO mice.

Previous studies indicate that ERSR is important in the mediation of tubular damage in AKI of different etiologies including those caused by cisplatin [44]. Based on our *in vitro* studies, which indicate that the up-regulation of polyamine catabolism leads to the activation of ERSR and onset of apoptosis, we examined the effect of modulation of polyamine catabolism on the activation of ERSR and onset of apoptosis in cisplatin AKI. To this end, we compared the expression of BiP and CHOP in the kidneys of control and cisplatin-treated Wt, SMOX-KO and SSAT-KO mice. The renal expression levels of BiP and CHOP were similar in the control samples from all 3 genotypes **(Fig 7A)**. The expression of BiP and CHOP increased in the kidneys of mice from all three genotypes after cisplatin treatment **(Fig 7A)**. However, the BiP and CHOP expression levels were reduced in the kidneys of cisplatin treated SMOX-KO and SSAT-KO mice compared to their cisplatin-treated Wt counterparts **(Fig 7A)**. Activated caspase 3 levels were also significantly elevated in the kidneys of Wt compared to those of SSAT-KO and SMOX-KO mice **(Fig 7B)**, suggesting that the presence of intact polyamine catabolic activity leads to the onset of more robust ER stress and apoptotic response.

**Fig 8:**
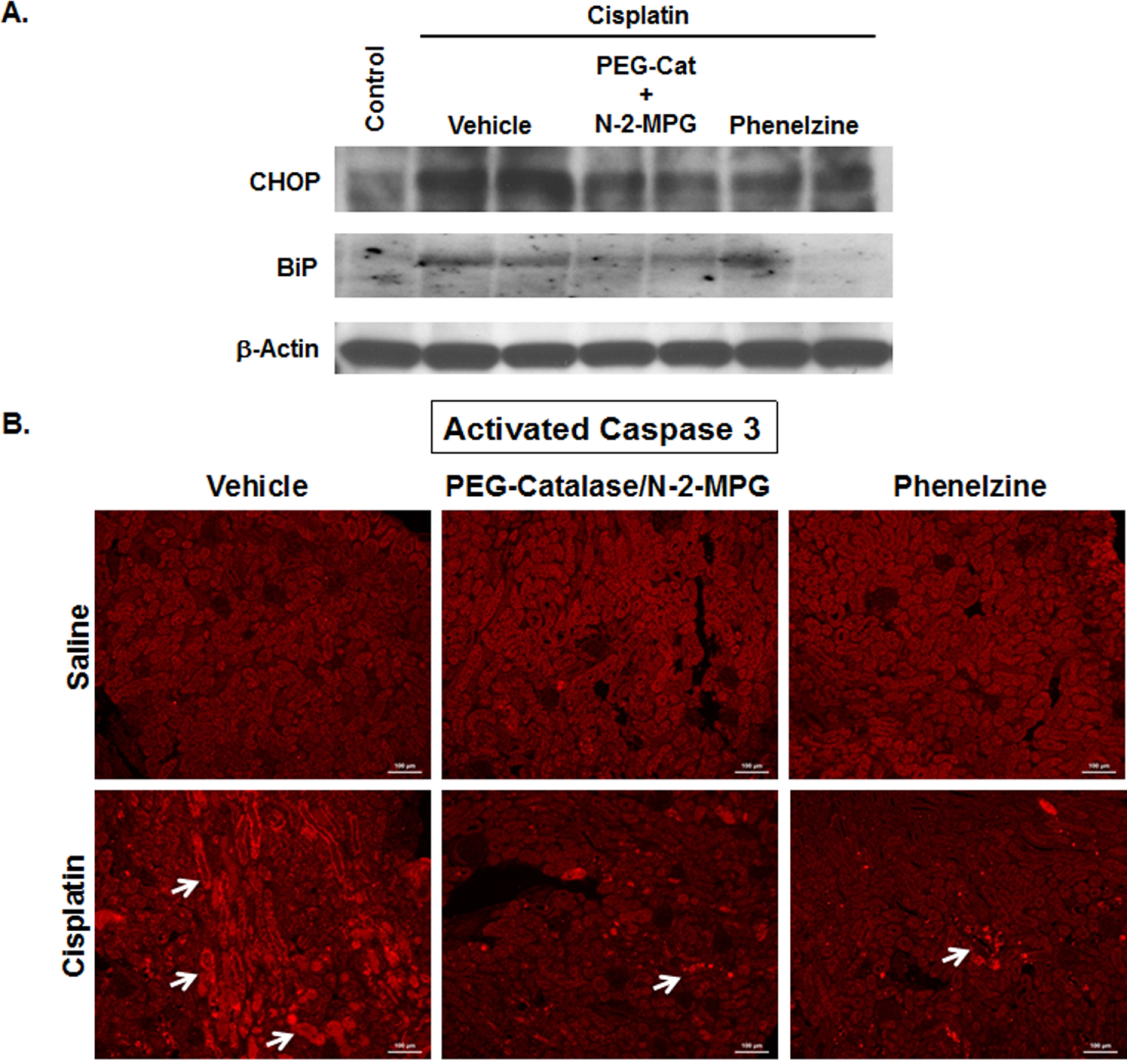
The impact of neutralization of toxic products of polyamine degradation on the induction of ERSR and onset of apoptosis. A) The expression levels of BiP and CHOP increased in the kidneys of cisplatin-treated mice compared to saline treated animals. The expression levels of BiP and CHOP were reduced in the kidneys of cisplatin-treated mice that were subjected to daily treatment with PEG-Cat/N-2-MPG or PLZ. The results are representative of 3 independent experiments. B) The activation of caspase 3 (arrows) was more robust in the kidneys of Wt cisplatin-treated mice receiving vehicle compared to the mice treated with cisplatin and subjected to daily PEG-Cat/N-2-MPG or PLZ treatment.

Products of polyamine degradation, H_2_O_2_ and aminoaldehydes, are known inducers of ER stress and apoptosis [39, 40]. Based on the ability of PEG-Cat/N-2-MPG and PLZ to modulate the severity of cisplatin AKI, we next determined whether the protective effect of these chemicals in cisplatin AKI is associated with the reduction of severity of ERSR. As demonstrated, the expression levels of BiP and CHOP increased in the kidneys of cisplatin-treated mice compared to saline treated animals **(Fig 8A)**. However, the expression levels of BiP and CHOP were significantly reduced in the kidneys of cisplatin-treated mice that were subjected to daily treatment with PEG-Cat/N-2-MPG or PLZ **(Fig 8A)**. Furthermore, the activation of caspase 3 was significantly more robust in the kidneys of Wt cisplatin-treated mice receiving vehicle compared to the mice treated with cisplatin and subjected to daily PEG-Cat/N-2-MPG or PLZ treatment **(Fig 8B)**.

The results of these experiments indicate that the severity of cisplatin AKI was associated with ERSR activation in Wt mice and was significantly modulated in SMOX-KO and SSAT-KO mice. Similarly, neutralization of polyamine degradation products diminishes the activation of ERSR.

## Discussion

In the present studies we tested the hypotheses that: 1) the expression levels of SSAT and SMOX increase in response to cisplatin treatment; 2) cisplatin-induced AKI is in part mediated via enhanced activity of polyamine catabolic enzymes and through generation of H_2_O_2_ and aminoaldehydes (e.g. 3-aminopropanal, acetyl-3-aminopropanal and acrolein); and 3) Increased polyamine catabolism activates ERSR, a pathway that is critical to the mediation of cell injury, tissue damage and organ dysfunction. Our results demonstrated that the expression and activity of polyamine catabolic enzymes, SSAT and SMOX, increase in kidneys of mice treated with cisplatin (Fig 2). Our results further revealed that the ablation of SSAT and SMOX genes reduces the severity of kidney dysfunction and tubular damage, and protects against cisplatin AKI (Fig 4). Examination of kidney polyamine levels indicated that deletion of SSAT and SMOX caused a reduction in Spm and Spd levels, respectively, under baseline conditions (Fig 3). The ablation of SSAT or SMOX blunted the increase in Put levels and modulated the alterations in Spm and Spd levels following treatment with cisplatin (Fig 3).

Products of polyamine degradation, 3-aminopropanal, acetyl- 3-aminopropanal and H_2_O_2_, are profoundly cytotoxic [11]. The degradation of H_2_O_2_ by cell-permeable PEG-catalase and/or neutralization or sequestration of aminoaldehydes by N-2-MPG or PLZ, respectively, reduced the severity of cisplatin AKI, as determined by reductions in serum creatinine levels and the extent of renal tubular damage (Fig 5).

H_2_O_2_, an important inducer of tissue damage in AKI, is generated as a result of alterations in mitochondrial activity as well as enhanced polyamine oxidation [11, 45]. Therefore, maneuvers that neutralize H_2_O_2_ constitute a more general protective treatment in AKI. On the other hand aminoaldehydes such as 3-aminopropanal, acetyl- 3-aminopropanal and acrolein are exclusively or predominantly generated as a result of polyamine oxidation [34, 46, 47]. The maladaptive roles of the aforementioned metabolites in the mediation of cerebral ischemia are well documented [34, 46, 47]. These biogenic amines accumulate in and disrupt the integrity of lysosomal membranes leading to the release of proteolytic enzymes; this in turn damages the mitochondria and induces apoptosis. Studies by Ivanova et. al. [30, 33] and Wood et. al. [34, 46, 47] indicate that 3-aminopropanal is a mediator of cell injury and that in a model of ischemic brain injury compounds that specifically target 3-aminopropanal impart protection against ischemia induced cerebral injury [34]. Coupled with the published reports, our data establish the role of aminoaldehydes produced as a result of degradation of polyamines as important mediators of tissue damage in injuries of varying etiologies in different organs.

Our data indicated that enhanced polyamine catabolism results in the activation of ERSR through generation of oxidative molecules (e.g. aminoaldehydes and H_2_O_2_) and/or a reduction in hypusinated-eIF5A levels consequent to polyamine depletion, and plays a critical role in cisplatin AKI. It has been demonstrated that depletion of polyamines reduces the hypusynation of elF5A, interferes with protein synthesis and leads to ER stress [10, 48]. Induction of ERSR in response to elevated levels of aldehydes and H_2_O_2_, products that are generated as a result of polyamine degradation, has also been demonstrated both *in vitro* and *in vivo* [39, 40]. Our previous studies indicated that the degradation of H_2_O_2_ by catalase modifies the severity of oxidative stress and reduces cellular damage in SSAT over-expressing cells [32].

The current work strongly suggests that the toxic byproducts of polyamine catabolism are important mediators of kidney injury in cisplatin-treated animals (Fig 5). Whether polyamine depletion per se (e.g. reduction in the inherent free radical scavenging properties of Spm) and reduced activity of eIF5A (through its reduced hypusination) are also important to cisplatin-induced renal injury could not be excluded. However, the protection against cisplatin AKI in SMOX KO mice, which exhibit similar alterations in tissue polyamine levels to that of Wt mice, argues against a major role for polyamine depletion in the mediation of kidney injury by cisplatin.

The induction of DNA damage, ERSR, mitochondrial dysfunction, growth arrest and apoptosis in SSAT over-expressing cells indicate that enhanced polyamine catabolism affects multiple pathways that are critical to the mediation of cell injury. Enhanced polyamine catabolism has also been demonstrated in I/R, infection, sepsis, toxic and traumatic insults in kidney, liver, gastrointestinal tract and brain [20–25]. The ablation or inhibition of enzymes involved in polyamine catabolism reduces the severity of renal and hepatic I/R and toxic injuries [23, 27–29]. Furthermore, a number of studies indicate that neutralization of toxic products of polyamine degradation can reduce the severity of ischemic brain injury [30, 33, 34, 46, 47].

The maladaptive role of polyamine catabolism and its byproducts in the mediation of tissue injury is well documented; however, the molecular mechanisms through which enhanced catabolism of polyamines contributes to the induction of tissue damage had not been elucidated. In the current studies we demonstrate that polyamine catabolism is enhanced in response to cisplatin treatment and leads to the onset of ERSR and induction of apoptosis consequent to the generation of toxic molecules. We propose that treatments aimed at blocking the catabolism of polyamines or enhancing the removal and/or sequestering of its toxic metabolites will provide significant protection against cisplatin induced AKI.

## Supporting information

S1 Fig Generation and genotyping of SMOX-KO mice. **A)** Diagram of the vector created for the generation of *Smox* knockout mice. A Neomycin marker (Neo) was introduced into the murine *Smox* gene by restriction digest. The addition of the Neo cassette also resulted in the removal of *Smox* exons IV, V, and VI resulting in a truncated sequence lacking the coding region for the catalytic domain in exon V. Blue rectangles represent exons; yellow rectangle within exon V represents the FAD binding region; green dashed lines indicated homologous recombination of the vector into the mouse genome. **B)** Genotyping of SMOX mice. Mice were genotyped as outlined in S1 Table. Mice that are wild-type (658bp) or homozygous *Smox*-KO (300bp) will have bands as above; heterozygous mice will have both bands.
(PDF)

S2 Fig Northern blot pictures for Fig 2. Uncropped pictures of northern blots (SSAT, SMOX and GAPDH) and ethidium bromide stained RNA gel (28s rRNA) for Fig 2.
(PDF)

S3 Fig Western blots for Fig 3. Uncropped pictures of western blots used in Fig 3.
(PDF)

S4 Fig Western blots for Fig 6. Uncropped pictures of western blots used in Fig 6.
(PDF)

S5 Fig Western blots for Fig 7. Uncropped pictures of western blots used in Fig 7.
(PDF)

S6 Fig Western blots for Fig 8. Uncropped pictures of western blots used in Fig 8.
(PDF)

S1 Table Primers for *Smox*-KO genotyping. Standard hot-start (95°C for 5-minutes) PCR conditions with a 5-minute extension (72°C) time (30 cycles: 95°C- 30 sec, 60°C- 30 sec, 72°C-5 minutes) were used for the amplification of genomic DNA. Mice that are wild-type (658bp) or homozygous *Smox*-KO (300bp) will have bands as above; heterozygous mice will have both bands (S1 Fig).
(PDF)
