# Supplementary figures and images for: Activation of endoplasmic reticulum stress response by enhanced polyamine catabolism is important in the mediation of cisplatin-induced acute kidney injury
Source: PLoS One. 2017 Sep 8;12(9):e0184570. doi: 10.1371/journal.pone.0184570 (PMC5590979; doi:10.1371/journal.pone.0184570)

# Supporting Information

S1 Fig.

A.

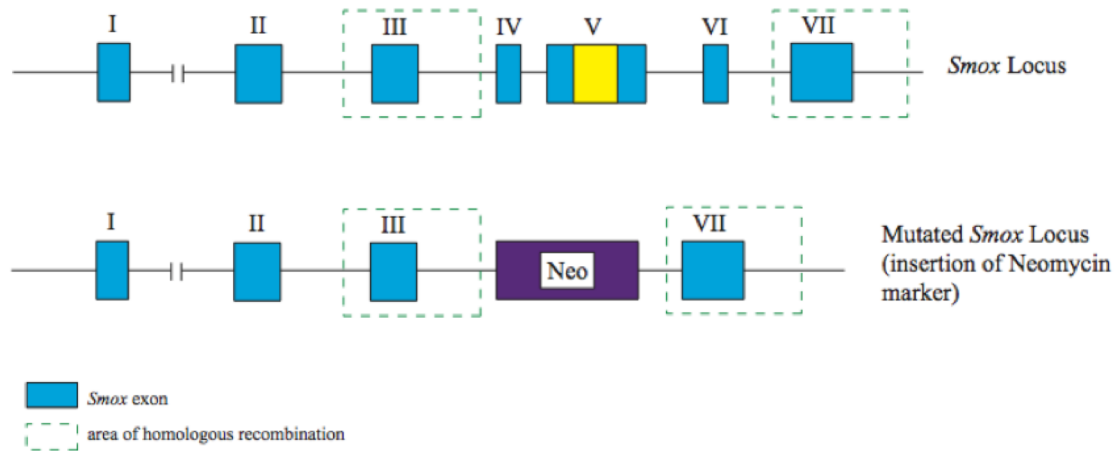

B.

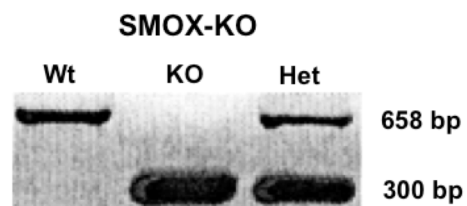

Supplement: S1 Fig — A) Diagram of the vector created for the generation of Smox knockout mice. A Neomycin marker (Neo) was introduced into the murine Smox gene by restriction digest. The addition of the Neo cassette also resulted in the removal of Smox exons IV, V, and VI resulting in a truncated sequence lacking the coding region for the catalytic domain in exon V. Blue rectangles represent exons; yellow rectangle within exon V represents the FAD binding region; green dashed lines indicated homologous recombination of the vector into the mouse genome. B) Genotyping of SMOX mice. Mice were genotyped as outlined in S1 Table. Mice that are wild-type (658bp) or homozygous Smox-KO (300bp) will have bands as above; heterozygous mice will have both bands. (PDF) [file pone.0184570.s001.pdf]

**S2 Fig. Northern blot pictures for Fig. 2.**

SSAT

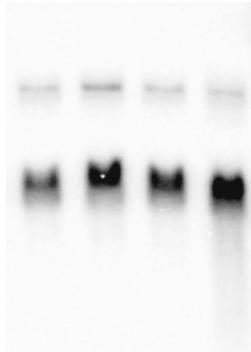

SMOX

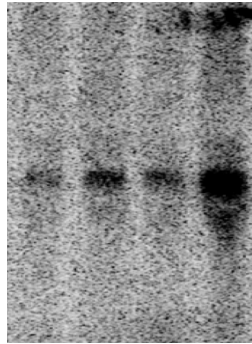

GAPDH

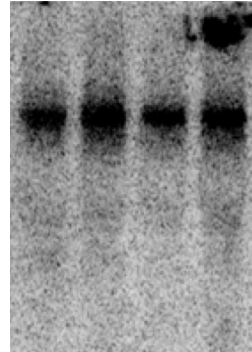

28s rRNA

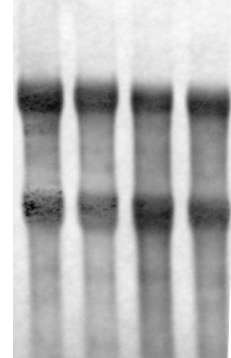

Supplement: S2 Fig — Uncropped pictures of northern blots (SSAT, SMOX and GAPDH) and ethidium bromide stained RNA gel (28s rRNA) for Fig 2. (PDF) [file pone.0184570.s002.pdf]

**S3 Fig. Western blots for Fig. 3**

SMOX

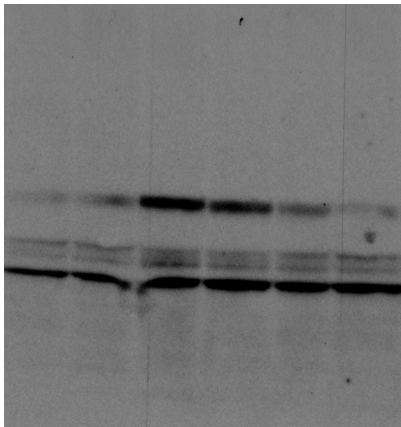

<SMOX (~62kDa)

$\beta$ -Actin

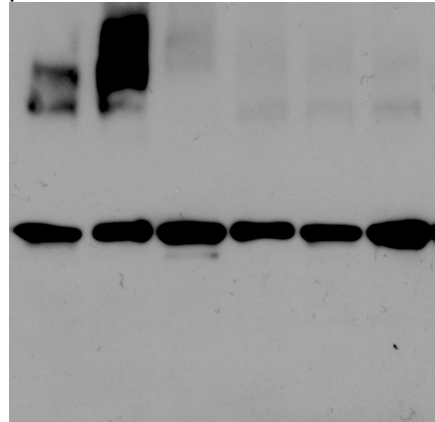

Supplement: S3 Fig — Uncropped pictures of western blots used in Fig 3. (PDF) [file pone.0184570.s003.pdf]

**S4 Fig. Western blots for Fig. 6.**

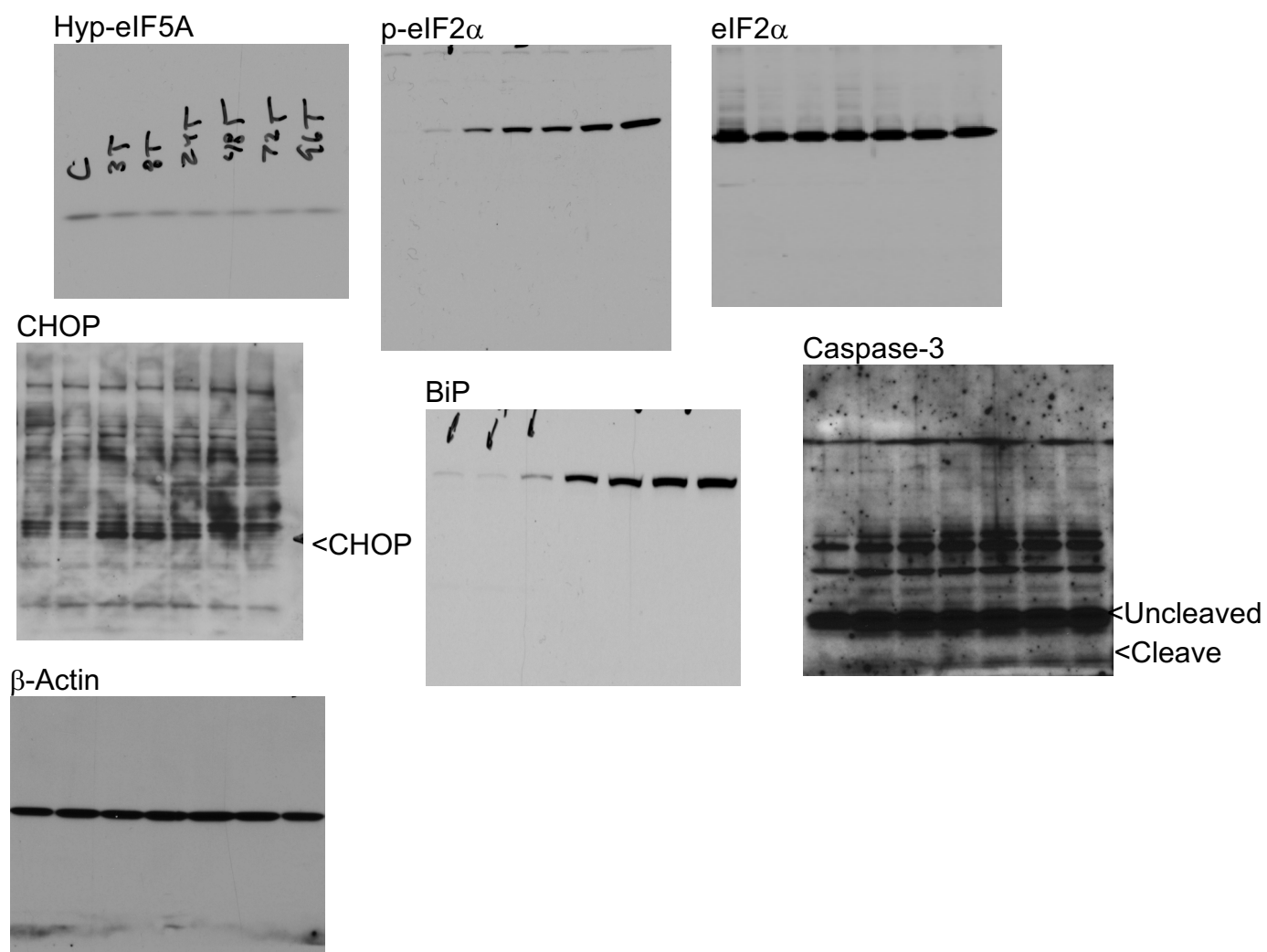

Supplement: S4 Fig — Uncropped pictures of western blots used in Fig 6. (PDF) [file pone.0184570.s004.pdf]

S5 Fig. Western blots for Fig. 7.

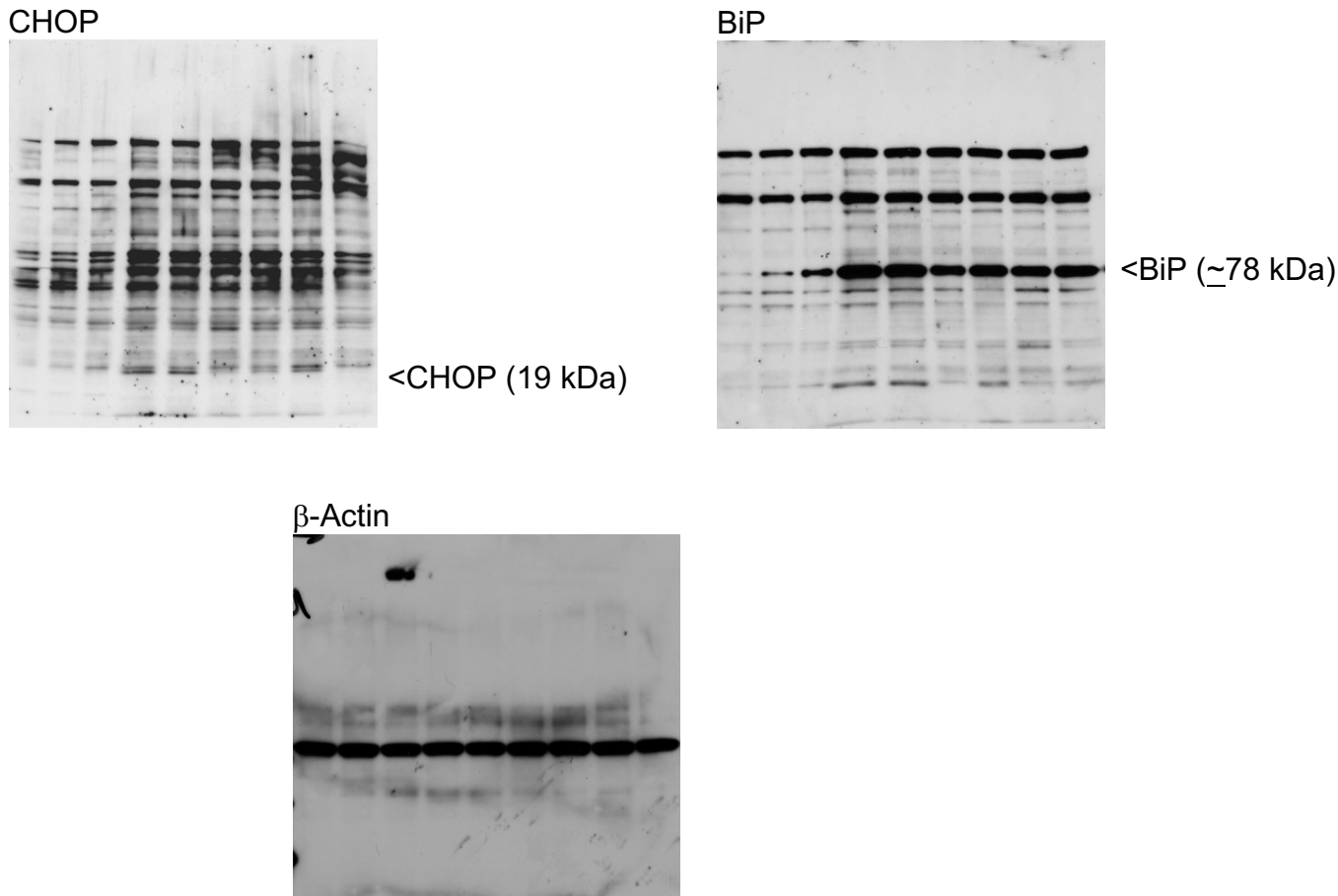

Supplement: S5 Fig — Uncropped pictures of western blots used in Fig 7. (PDF) [file pone.0184570.s005.pdf]

S6 Fig. Western blots for Fig. 8.

CHOP

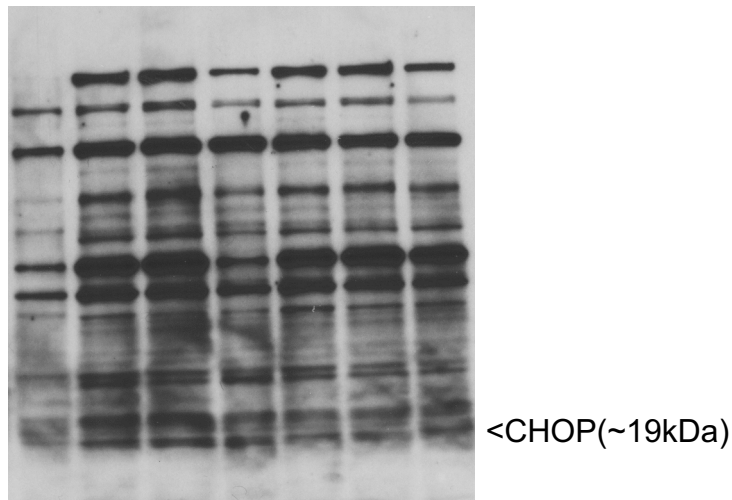

BiP

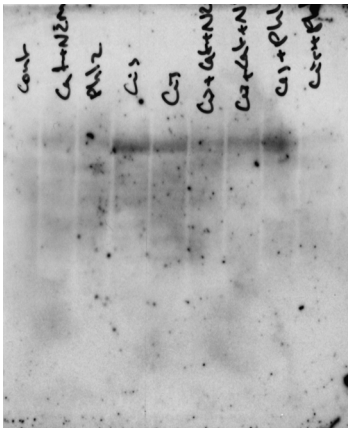

$\beta$ -Actin

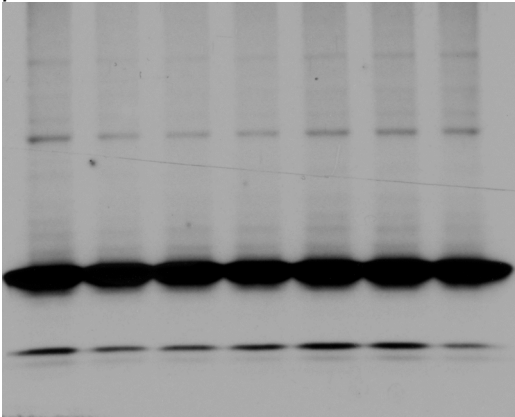

Supplement: S6 Fig — Uncropped pictures of western blots used in Fig 8. (PDF) [file pone.0184570.s006.pdf]
