# Supplementary material for: Activation of endoplasmic reticulum stress response by enhanced polyamine catabolism is important in the mediation of cisplatin-induced acute kidney injury
Source: PLoS One. 2017 Sep 8;12(9):e0184570. doi: 10.1371/journal.pone.0184570 (PMC5590979; doi:10.1371/journal.pone.0184570)
Supplement: S1 Table — Standard hot-start (95°C for 5-minutes) PCR conditions with a 5-minute extension (72°C) time (30 cycles: 95°C- 30 sec, 60°C- 30 sec, 72°C-5 minutes) were used for the amplification of genomic DNA. Mice that are wild-type (658bp) or homozygous Smox-KO (300bp) will have bands as above; heterozygous mice will have both bands (S1 Fig). (PDF) [file pone.0184570.s007.pdf]

S1 Table. Primers for *Smox*-KO genotyping.

Primer Target	Forward Sequence	Reverse Sequence	Fragment size
Smox insert	5' TTGGAGCGGCCCTCTCTTCTGTCC 3'	5' CTTTCCCGCCCTCCCTTGCGCTAC 3'	~300 bp
Smox wild-type	5' GGTCACTGTGGATTAAGGAGTGGG 3'	5' GGTCATCCCTGATGCGATTCCGCA 3'	~658 bp
